# Peripheral Blood Cell Signatures of *Plasmodium falciparum* Infection during Pregnancy

**DOI:** 10.1371/journal.pone.0049621

**Published:** 2012-12-11

**Authors:** Samad Ibitokou, Mayke Oesterholt, Laurent Brutus, Sophie Borgella, Carine Agbowaï, Sèm Ezinmègnon, John Lusingu, Christentze Schmiegelow, Achille Massougbodji, Philippe Deloron, Marita Troye-Blomberg, Stefania Varani, Adrian J. F. Luty, Nadine Fievet

**Affiliations:** 1 Centre d'étude et de recherche sur le paludisme associé à la grossesse et à l'enfance (CERPAGE), Faculté des Sciences de la Santé, Université d'Abomey-Calavi, Cotonou, Benin; 2 Institut de Recherche pour le Développement, UMR 216, Mère et enfant face aux infections tropicales, Paris, France; 3 PRES Sorbonne Paris Cité, Faculté de Pharmacie, Université Paris Descartes, Paris, France; 4 Department of Medical Microbiology, Radboud University Nijmegen Medical Centre, Nijmegen, The Netherlands; 5 National Institute for Medical Research, Tanga, Tanzania; 6 Centre for Medical Parasitology, Institute of International Health, Immunology, and Microbiology, University of Copenhagen and Department of Infectious Diseases, Copenhagen University Hospital, Copenhagen, Denmark; 7 Department of Immunology, Wenner-Gren Institute, Stockholm University, Stockholm, Sweden; 8 Unit of Microbiology, Department of Hematology and Oncology, University of Bologna, Bologna, Italy; Instituto Gulbenkian de Ciência, Portugal

## Abstract

Sequestration of *Plasmodium falciparum*-infected erythrocytes in placental intervillous spaces causes inflammation and pathology. Knowledge of the profiles of immune cells associated with the physiopathology of pregnancy-associated malaria (PAM) is scarce. We conducted a longitudinal, prospective study, both in Benin and Tanzania, including ∼1000 pregnant women in each site with systematic follow-up at scheduled antenatal visits until delivery. We used *ex vivo* flow cytometry to identify peripheral blood mononuclear cell (PBMC) profiles that are associated with PAM and anaemia, determining the phenotypic composition and activation status of PBMC in selected sub-groups with and without PAM both at inclusion and at delivery in a total of 302 women. Both at inclusion and at delivery PAM was associated with significantly increased frequencies both of B cells overall and of activated B cells. Infection-related profiles were otherwise quite distinct at the two different time-points. At inclusion, PAM was associated with anaemia, with an increased frequency of immature monocytes and with a decreased frequency of regulatory T cells (Treg). At delivery, infected women presented with significantly fewer plasmacytoid dendritic cells (DC), more myeloid DC expressing low levels of HLA-DR, and more effector T cells (Teff) compared to uninfected women. Independent associations with an increased risk of anaemia were found for altered antigen-presenting cell frequencies at inclusion, but for an increased frequency of Teff at delivery. Our findings emphasize the prominent role played by B cells during PAM whenever it arises during pregnancy, whilst also revealing signature changes in other circulating cell types that, we conclude, primarily reflect the relative duration of the infections. Thus, the acute, recently-acquired infections present at delivery were marked by changes in DC and Teff frequencies, contrasting with infections at inclusion, considered chronic in nature, that were characterized by an abundance of immature monocytes and a paucity of Treg in PBMC.

## Introduction

Pregnancy is characterized by still generally poorly defined changes in the immunological equilibrium needed to protect the mother and the fetus from invading pathogens whilst at the same time tolerating the highly immunogenic paternal alloantigens in order to sustain fetal integrity. Through their capacity to modulate immunological responses, maternally-derived regulatory T cells (Treg) are now thought to play a pivotal role in the tolerance of the fetus by the mother's immune system, a role reflected by their reportedly dramatic increase in numbers during pregnancy [1]–[4]. Dendritic cells (DC), particularly those DC located in the decidual tissues, are central controllers of the materno-foetal tolerance process through their overall influence, governed by the presence of Treg, on immune responses in general [3]. A further level of maternal-foetal tolerance extends to the expression by fetal trophoblasts of non-classical human leucocyte antigens (HLA) class I molecules, such as HLA-G. Such molecules do not trigger the natural killer (NK) cell-mediated cytotoxic response elicited by abnormal expression of HLA molecules that commonly occurs on cells that are stressed or infected [3]. For obvious reasons, the knowledge we have of such aspects is derived from examination of placental tissues at delivery and/or of peripheral blood, with the latter providing the only accessible ‘window’ through which one can view changes in cell numbers and phenotypes as a function of gestational age. Indeed, data from recently conducted longitudinal studies have revealed increasing evidence of significant changes in both the quantity and the quality of Treg, DC and other cell types during normal pregnancies in high-income countries [5]–[8].

Infections during pregnancy can represent profound disturbances to the delicate materno-foetal equilibrium, especially infections that are localised to the placenta itself. In the public health context of low-income countries, one of the most prominent and important examples of such an infection is, without doubt, *Plasmodium falciparum*, the protozoan parasite that causes malaria. Its prominence as such reflects the burden of maternal and neonatal disease and death for which it is directly responsible worldwide [9]. The clinical and pathological outcomes include maternal anaemia, premature birth and low birthweight, which are a direct consequence of infection of the placenta by *P. falciparum* [10]. That tissue specificity arises from the adhesive interactions between at least one parasite-derived protein, referred to as VAR2CSA, that is inserted into the membrane of *P. falciparum*-infected erythrocytes (*Pf*iE), and a host receptor, chondroitin sulphate A (CSA), that is expressed on syncytiotrophoblasts [11]. Those interactions lead to accumulations of *Pf*iE in the intervillous spaces of the placenta that are accompanied by an inflammatory response, predominantly involving monocytes and to some extent also B and NK cells and neutrophils [12], [13]. Numerous published studies have documented the nature of the immune response elicited by placental infection with *P. falciparum*, using peripheral venous and/or placental blood collected at delivery. Such studies have quantified levels of cytokines and chemokines in plasma, and have also identified the phenotypes of the cells as well as non-specific and parasite-specific response profiles, following stimulation *in vitro*, of mononuclear cell populations [14]–[16]. A single study has documented specific infection-related alterations in DC populations at delivery [17]. There are, however, no published reports of similar data collected longitudinally during pregnancy in women with and without *P. falciparum* infection. The study presented here is therefore a first step in the attempts to fill this large gap in our knowledge. Within the overall framework of the STOPPAM project, the study's primary objective was thus to evaluate the impact of pregnancy-associated malaria (PAM) on the phenotypic composition and activation status of peripheral blood mononuclear cells (PBMC), and to attempt to identify PBMC profiles that are associated with particular outcomes e.g. maternal anaemia, in order to better understand the pathogenesis of PAM. As such, we designed the study to provide two ‘windows’ through which to observe cellular profiles in women with or without infection by *P. falciparum*, the first at inclusion into the study (during the second trimester for the majority of participants) and the second at delivery. Identical procedures were used, based on standardized flow cytometric staining methods with PBMC *ex vivo*, in two geographically separated study sites in sub-Saharan Africa, one in Benin and the other in Tanzania, that differ distinctly with respect to the patterns of transmission of malaria. In both sites, detailed clinical and parasitological data were collected from each participant at inclusion into the study and thereafter throughout pregnancy up to and including delivery. The resulting databases thus allow for in-depth assessments of outcomes related to the range of immunological variables evaluated.

## Materials and Methods

### Ethics statement

The STOPPAM study received ethical clearance from the ethics committees of the Health Science Faculty of the University of Abomey-Calavi, Benin, and of the National Institute for Medical Research of Tanzania.

### Study population

The study populations comprise sub-groups drawn from the cohorts of pregnant women that participated in a longitudinal study known as “Strategies TO Prevent Pregnancy Associated Malaria” (STOPPAM) that was conducted in parallel in the two study sites i.e. Benin and Tanzania. After giving written informed consent, ∼1000 pregnant women at ≤24 weeks' gestational age were included both in Comé, located in the Mono province 70 km west of Cotonou, the economic capital of Benin, and in Korogwe, located in the Tanga Region of north-eastern Tanzania. Transmission of malaria in the area of the Beninese study site is considered as moderate-high (the entomological inoculation rate (EIR) was 20.5 in neighbouring Tori Bossito) [18], whilst in the Korogwe area transmission was, historically, high (EIR = 90) [19] but has recently declined sharply [20]. Perennial transmission with seasonal peaks characterizes both sites. The STOPPAM study design has been described in detail elsewhere [21]. Briefly, ultrasound examinations were used to determine gestational age, and women were followed from inclusion up to and including delivery through a series of scheduled ante-natal visits (ANV) during which ultrasonographic, clinical and parasitological assessments were conducted. Women were also encouraged to attend the clinic (‘emergency’ visits) in the event of any perceived illness or other pregnancy-related problem. At all visits (ANV or emergency), infection with *P. falciparum* was identified through the use of rapid diagnostic tests (RDT), and those with a positive RDT were given appropriate anti-malarial treatment. Retrospective parasitological confirmation of infections comprised microscopical examination of routinely prepared, giemsa-stained thick and thin blood smears. All women received two standard curative treatment doses, spaced at least 1 month apart, of sulphadoxine-pyrimethamine according to the national policies for intermittent preventive treatment in pregnancy (IPTp). The sub-groups selected for cellular immunological studies both at inclusion and at delivery described here were identified on the basis either of their current or their past infection status, with a case-control design. At inclusion, on the basis of an informed estimate of a prevalence of 10%, we expected 100 women to present with infection with *P. falciparum*. For immunological assessments at inclusion we therefore planned to include a total of 200 women, comprising 100 ‘cases’ of women infected with *P. falciparum* and 100 ‘controls’ of uninfected woman matched to ‘cases’ by age, gravidity and gestational age. Cases were recruited sequentially according to their inclusion into the overall study, with the matched uninfected controls selected and recruited as soon as practically possible after each case. At delivery infected women (‘cases’ who may or may no have been infected earlier) were again included into the immunological sub-study chronologically, but 2 different uninfected ‘control’ sub-groups, matched by age and gravidity to cases, were defined using the detailed clinical and parasitological histories available as a result of the follow-up: (i) an uninfected group comprising those with no evidence of infection with *P. falciparum* at inclusion or subsequently throughout follow-up or at delivery, (ii) an exposed group comprising those uninfected at delivery but who had at least one infection episode at inclusion or during follow-up. In each site, a majority of those selected at inclusion and delivery were different individuals although samples from a minority were included in both. The low prevalence of *P. falciparum* infection in Tanzanian women resulted in markedly lower-than-expected numbers for inclusion in sub-groups at both time-points. Data from women in either study site with proven seropositivity for HIV or with unknown HIV sero-status were not included in the analyses.

### P. falciparum parasites detection in blood smears

Plasmodial parasite detection in freshly-drawn blood was performed using a rapid diagnostic test (RDT) (Parascreen*, Zephyr Biomedical Systems, Goa, India), and retrospectively through standard high-power microscopical examination of thin and thick blood smears prepared from peripheral blood and of placental impression smears.

### Blood collection and cell preparation

Peripheral venous blood samples were collected at inclusion and at delivery in vacutainers containing citrate phosphate dextrose adenine (CPDA) anticoagulant. For the detailed immunological studies described here, samples were taken from 131 and 111 Beninese women at inclusion and at delivery, and from 38 and 27 women in the Tanzanian cohort, respectively. The blood samples were transported to the research laboratories in the respective study sites and were processed for immunological assessments within 4 hours. Peripheral blood mononuclear cells (PBMC) were isolated using Leucosep tubes (Greiner-Bio) according to the manufacturer's description and were subsequently used for the immunophenotyping described below.

### Dendritic cell, B cell and monocyte immunophenotyping

**Figure 1:**
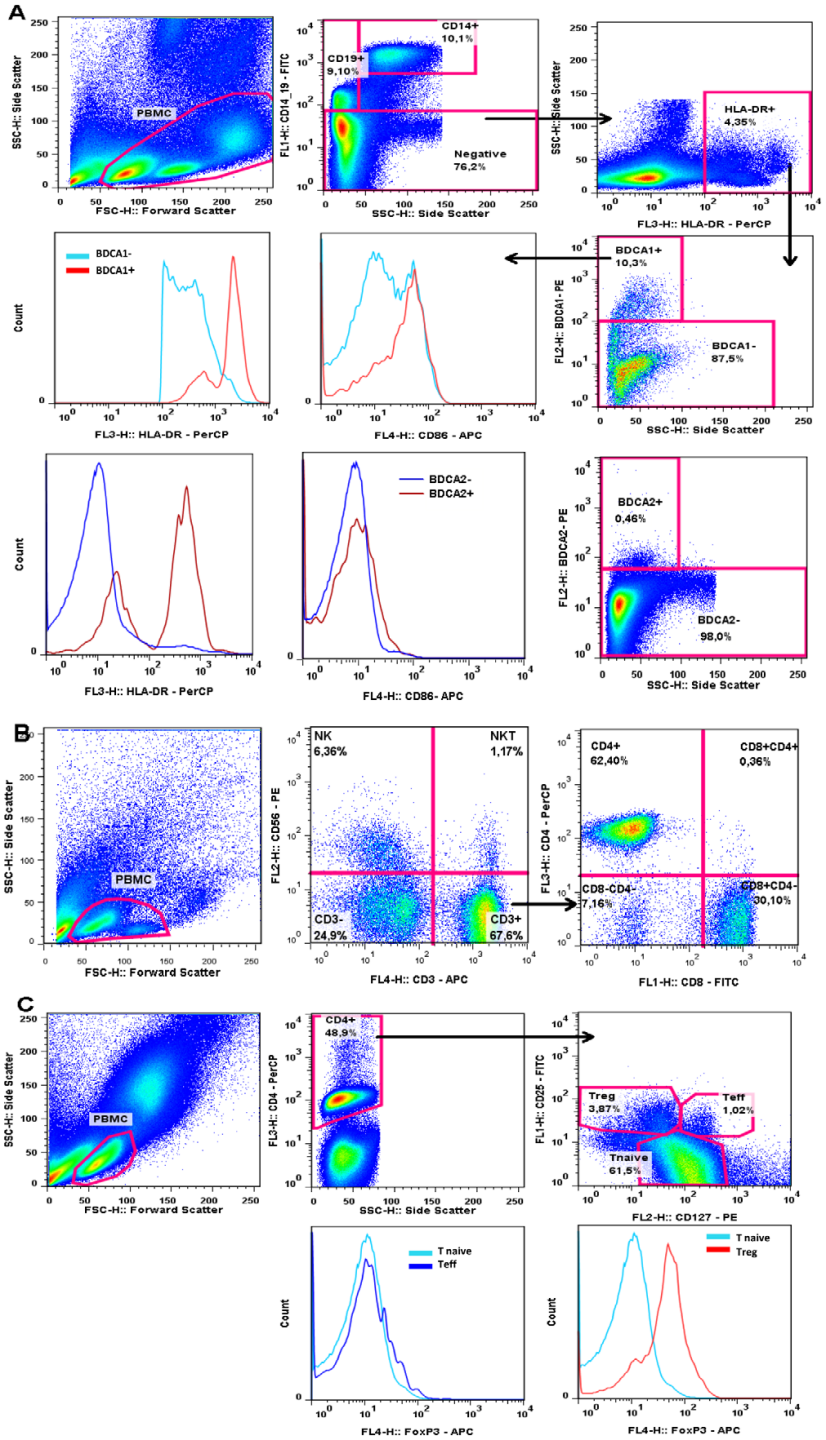
Cytometry-based gating for definition of phenotypes. (A) Monocytes (CD14^+^), B cells (CD19^+^) were gated from PBMC. pDC were directly gated from the CD14^−^CD19^−^ population whilst mDC were gated from the CD14^−^CD19^−^HLA-DR^+^ population. CD86 and HLA-DR expression were determined by MFI (Mean Fluorescence Intensity). (B) NK, NK T and CD3^+^ cells were gated from PBMC. CD4^+^ and CD8^+^ T cells were gated from CD3^+^. The gating strategies for Treg (CD4^+^CD25^+^CD127^−^) and Teff (CD4^+^CD25^+^CD127^+^) are presented in (C). Cell frequencies were determined as a percentage of PBMC, and relative FoxP3 expression level determined as a function of FoxP3 expresssion by naïve CD4^+^ T cells (CD4^+^CD25^−^).

PBMC were washed in staining buffer (PBSx1, EDTA 5 mM, 2% FBS) for 10 min at 150 g and resuspended at a concentration of 10 million cells/ml. Cells were then incubated with 10 µl of FcR Blocking reagent (Miltenyi Biotec, Gladbach, Germany) for 10 minutes in the dark at 4°C to prevent non-specific labelling. Specific surface labelling was then performed by adding anti-BDCA-1-Phycoerythrin (PE) for myeloid dendritic cells (mDC) and anti-BDCA-2-PE for plasmacytoid DC (pDC) detection (all Miltenyi Biotec), anti-CD14-Fluorescein isothiocyanate (FITC) for monocytes, anti-CD-19-FITC for B cells, and the combination of anti-HLA-DR-Peridinin chlorophyll protein (PerCP) and anti-CD86-Allophycocyanin-cyanin (APC) (all BD Pharmingen, San Diego, CA) antibodies for cells' activation status. After incubating for 30 minutes at 4°C in the dark, cells were then washed and fixed with FACS lysing solution (BD Pharmingen). The cells were finally resuspended in 300 µl of staining buffer, acquired using BD FACSCalibur and analyzed using CellQuest Pro or FlowJo 7.6 software. Gating strategies are shown in Figure 1A.

### T cell, NK cell and T regulatory cell immunophenotyping

Cells were resuspended and incubated with 10 µl of FcR Blocking reagent (Miltenyi Biotec) for 10 minutes in the dark at 4°C. Cells were then incubated for 30 minutes in the dark, with specific antibodies. Anti-CD3-APC, anti-CD8-FITC, anti-CD4-PerCP and anti-CD56-PE (BD Pharmingen) were used for T lymphocyte and NK cell labelling, whilst anti-CD25-FITC and anti-CD127-PE (BD Pharmingen) were used for regulatory T cell (Treg) labelling. Anti FoxP3-APC (BD Pharmingen) was added for Treg labelling according to the manufacturer's recommendations after permeabilisation and fixation with PermFix (BD Pharmingen). Cells were acquired in 300 µl of PBS 3% FBS using BD FACSCalibur and analyzed by CellQuest Pro or FlowJo 7.6 software. Gating strategies are shown in Figure 1B & C.

### Data analysis

When using matching for inclusion of controls our original intention was to enhance statistical power through the use of paired tests. In practice, resource restrictions precluded collection of samples from the intended number of women. In addition, the implementation of various exclusion criteria (HIV infection, missing information and/or data) resulted in an overall reduction in sample sizes and a consequently reduced number of ‘pairs’. In order to maximize the use of available data, we therefore chose to apply unpaired tests throughout, and retained gravidity, gestational age and age - the specific criteria used for matching - as variables in all analyses. The decline in transmission of malaria in the Tanzanian site resulted in a markedly lower prevalence of infection compared with Benin, and hence smaller sub-group sizes. Although it therefore lacked the same level of statistical power we nevertheless opted to maximize the use of the hard-won Tanzanian dataset to validate, where possible, the findings from Benin.

Data analysis was performed using STATA/MP 11.2 (StataCorp, College Station, TX USA) and Prism 5.0 (Graph pad Inc). For each continuous biological variable, the median of the distribution for uninfected women was used as the threshold value for the transformation of the variable into categorical variable (i.e. below or above the threshold). Categorical variables were compared with chi2 (÷^2^) or Fisher's exact tests and continuous variables compared with non-parametric tests (Mann Whitney). Multiple logistic, ordered logistic and linear regressions were performed using respectively “logit”, “ologit” and “regress” commands in STATA in order to identify biological variables associated with the risk for or the level of *P. falciparum* infections among pregnant women both at inclusion and at delivery in the Beninese cohort. The threshold value of statistical significance in univariate analyses for inclusion of variables into multivariate analyses was set at p<0,2. Results of analyses of data from Benin were confirmed in a multiple logistic regression by using data from the Tanzanian cohort as an external set of validation. In order to assess whether any of the biological variables measured were independently associated with the risk of anaemia at inclusion and/or at delivery, similar procedures to those described above were used. A prospective evaluation of the association between biological variables at inclusion and anaemia at delivery was also conducted using similar procedures for selection.

## Results

### Demographic and other characteristics of the study populations

In order to compare data between *P. falciparum*-infected and uninfected pregnant women, sub-groups were selected from within the whole STOPPAM cohort in each site. In Benin the sub-groups selected comprised 131 women at inclusion (62 infected and 69 uninfected) and 111 at delivery (37 with infection at delivery, 27 ‘exposed’ women who had been infected at least once during pregnancy but were uninfected at delivery, 47 uninfected throughout pregnancy, Tables 1 & 2). In Tanzania, the sub-group at inclusion comprised 38 women (20 infected and 18 uninfected), whilst at delivery it comprised 27 women (9 infected at delivery, 7 ‘exposed’ and 11 uninfected throughout pregnancy, Table 2). To assess whether the women included in the sub-groups for immunological analysis were representative, relevant parameters were compared between them and the whole study cohort. As expected because of the bias inherent to the criteria used for their selection at inclusion (*P. falciparum* infections are more common in primigravidae, who are by definition younger), the sub-group of 131 Beninese women was of significantly lower gravidity and was significantly younger than the whole cohort (Table 1), but at delivery no such differences were apparent (Table 1). The smaller size of the Tanzanian cohort meant that similar comparisons of the sub-groups were not meaningful.

**Table 1:** Characteristics of sub-groups compared with the whole cohort in Benin at inclusion and at delivery.

	Inclusion					Delivery				
Variables	Whole cohort		Sub-group			Whole cohort		Sub-group		
	n		n		p*	n		n		p*
**Gestational age in weeks**	982	18.1 (5.0)	131	17.7 (4.1)	0.31	623	39.5 (2.3)	111	39.5 (1.5)	0.75
**Gravidity**	1037	3.4 (2.0)	131	3.0 (1.9)	0.02	630	3.5 (2.1)	111	3.3 (1.9)	0.25
**Age in years**	1023	26.4 (6.2)	131	25.2 (6.1)	0.04	618	26.7 (6.3)	110	26.2 (5.9)	0.50
**% possessing a bednet**	1037	32.1	131	28.2	0.37	630	29.5	111	34.2	0.32
**% with haemoglobin <11 g/dl**	1029	60.7	130	66.9	0.17	578	46.4	102	40.2	0.25
**Parasites/µL**	176	1538 (299)	59†	2399 (699)	0.19	70	15266 (1142)	33‡	13627 (1250)	0.83

Values are means (standard deviation) except for parasitaemia which are medians (interquartile ranges).

* Student t test or χ^2^ for proportions.

† 3 subjects were positive by RDT but negative by microscopy.

‡ 4 subjects were positive by RDT but negative by microscopy.

**Table 2:** Univariate analysis of sub-groups segregated according to the presence or absence of *P. falciparum* infection at inclusion and at delivery.

	Inclusion						Delivery							
	Benin			Tanzania			Benin				Tanzania			
Variables	Infected	Uninfected	p	Infected	Uninfected	p	Infected	Exposed	Uninfected	p	Infected	Exposed	Uninfected	p
	(n = 62)	(n = 69)		(n = 20)	(n = 18)		(n = 37)	(n = 27)	(n = 47)		(n = 9)	(n = 7)	(n = 11)	
**Gestational age in weeks**	17.4 (4.0)	17.9 (4.2)	0.44*	18.0 (2.7)	18.4 (3.6)	0.76*	39.3 (1.9)	39.8 (1.3)	39.6 (1.3)	0.35‡	39.4 (1.1)	39.5 (1.0)	38.9 (3.0)	0.80‡
**Gravidity**	2.9 (1.9)	2.9 (2.0)	0.97*	2.2 (1.8)	2.2 (0.8)	0.26*	3.3 (2.2)	2.6 (1.1)	3.7 (2.0)	0.10‡	3.2 (1.3)	1.7 (0.7)	2.8 (1.5)	0.07‡
**Age in years**	24.2 (5.9)	26.1 (6.2)	0.08*	23.3 (4.6)	24.0 (5.3)	0.32*	25.6 (6.5)	24.0 (4.2)	28.1 (5.7)	0.01‡	28.3 (7.3)	23.3 (4.1)	25.4 (6.3)	0.28‡
**% possessing a bednet**	19.3	36.2	0.03**	70.0	66.7	1.0**	29.7	25.9	42.5	0.20†	55.6	57.1	81.8	0.21†
**% with Hb <11 g/dl**	80.3	55.1	<0.01**	60.0	33.3	0.12**	53.1	44.0	28.9	0.03†	50.0	71.4	45.4	0.78†
**Parasites/µL**	2399 (345)	-	-	1107 (71)	-	-	12492 (1035)	-	-	-	4008 (1460)^a^	-	-	-

Values are means (standard deviation) except for parasitemia which are medians (interquartile ranges).

* Student t test,

** χ^2^ for proportions,

† Tendency test,

‡ Anova test.

a data from 2 subjects with parasitaemia.

We wished to assess the influence of *P. falciparum* infection on multiple parameters, and therefore used univariate analysis in a first step to make comparisons within the sub-groups. In Benin, women infected with *P. falciparum* at inclusion showed a non-significant trend to be younger, whilst a significantly higher proportion were anaemic and significantly fewer declared possessing a bednet compared with the uninfected group (Table 2). The same set of parameters did not differ significantly between the sub-groups of Tanzanian women at inclusion, although the proportion of those infected who were also anaemic was almost double that in the uninfected group (Table 2). At delivery, we wished to make as detailed a comparison as possible and therefore used the information generated by the longitudinal surveillance of women through pregnancy to identify 3 different groups of women based on their *P. falciparum* infection histories since inclusion into the study, namely (i) those never found infected from inclusion through to delivery (including at emergency clinic visits outside scheduled ANV), (ii) an ‘exposed’ group comprising those infected at least once during pregnancy but who were uninfected at delivery and (iii) those infected at delivery with or without recorded infection during pregnancy. We found that a higher proportion of infected mothers in Benin were younger and anaemia was more common amongst them compared with the uninfected group (Table 2). A separate logistical regression analysis, using dichotomised data for the sub-group at delivery to compare the infected group with all women found to be uninfected at delivery (i.e. both exposed and uninfected groups combined together), revealed a higher proportion of primigravidae in the infected group (data not shown). No such differences were seen at delivery in the Tanzanian mothers but the small sample sizes are restrictive in this case (Table 2).

### P. falciparum infection-related changes in the peripheral blood mononuclear cell composition at inclusion

The principal focus of this study concerned the effects of infection with *P. falciparum* during pregnancy on PBMC profiles. At inclusion into such a study, when access to placental blood is not practicable, it cannot be determined with absolute certainty that an infection detected in peripheral blood extends to the placenta. Current knowledge indicates that the state of placental vascularization first allows access to intervillous spaces by maternal blood, and hence by *P. falciparum*-infected erythrocytes (*Pf*iE), between the 10^th^–12^th^ weeks of gestation. Our own findings concerning VAR2CSA expression, nevertheless, suggest that *Pf*iE do indeed have access to cells expressing CSA (i.e. of placental origin) before the start of the 2^nd^ trimester in >90% of women (Tuikue Ndam N., unpublished observations). Within the selected sub-group of women in Benin there were eight (8) infected women with a gestational age (GA) <12 weeks at inclusion. Data from these women were not excluded from the analysis presented here.

**Figure 2:**
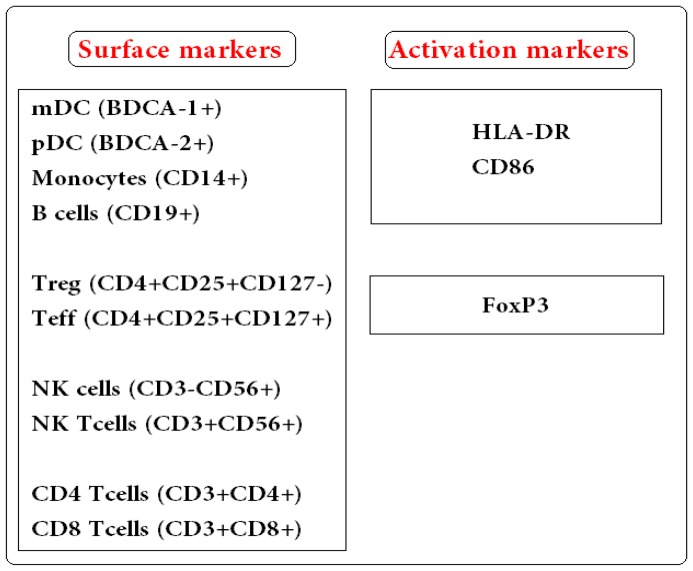
Basic flow cytometry-based phenotypic characterization of different cell populations investigated. The left panel lists the cell types identified with different surface markers and the right panel lists the separate markers used to characterize antigen-presenting cells activation status (HLA-DR & CD86) and to distinguish CD4^+^ regulatory T cells (Treg, expressing the transcription factor FoxP3) from effector T cells (Teff).

**Figure 3:**
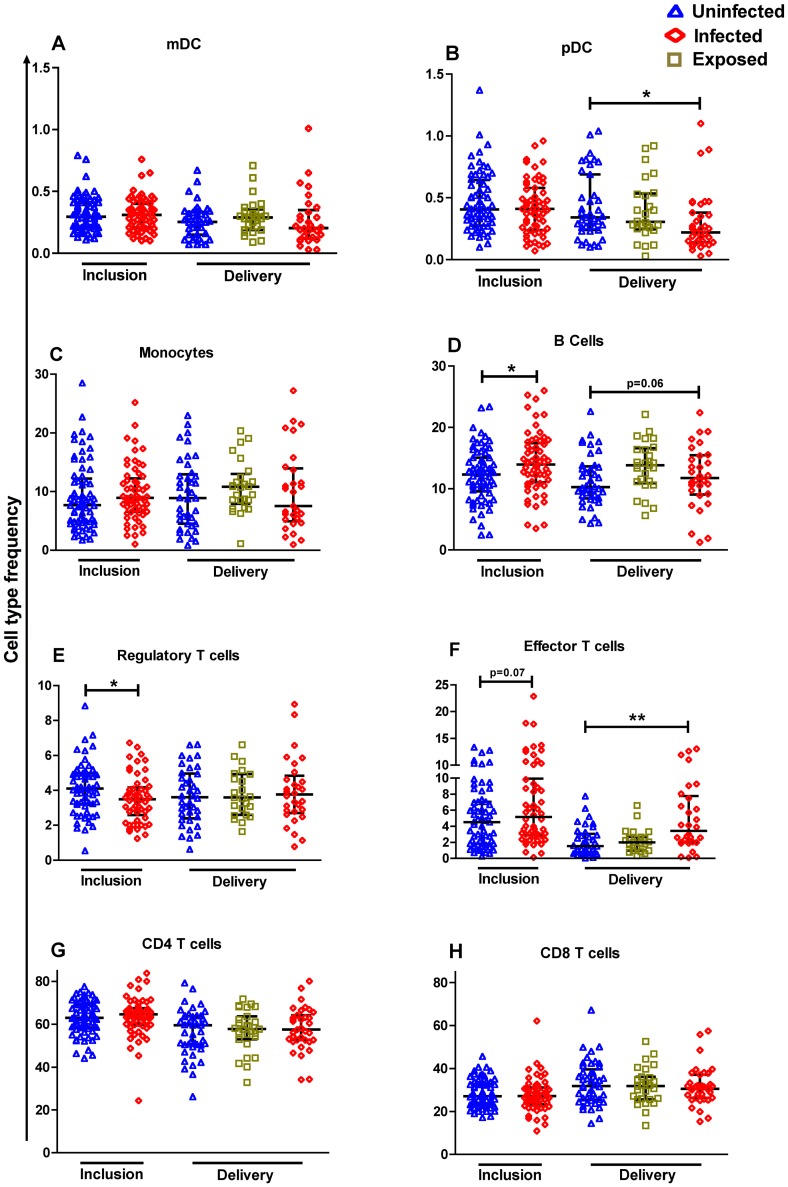
*P. falciparum* infection-related changes in peripheral blood mononuclear cell frequencies of pregnant Beninese at inclusion and at delivery. Scatter plots include bars depicting medians with interquartile ranges of antigen-presenting cells (A, B, C and D) and T cell subset frequencies (E, F, G and H) from 69 uninfected compared to 62 infected women at inclusion, and from 47 uninfected, 27 exposed and 37 infected women at delivery. The statistical significance of differences between profiles segregated according to the presence or absence of infection were determined using the non-parametric Mann Whitney U test for data at inclusion combined with the non-parametric Kruskall Wallis test for data at delivery. *p<0.05, **p<0.01.

**Figure 4:**
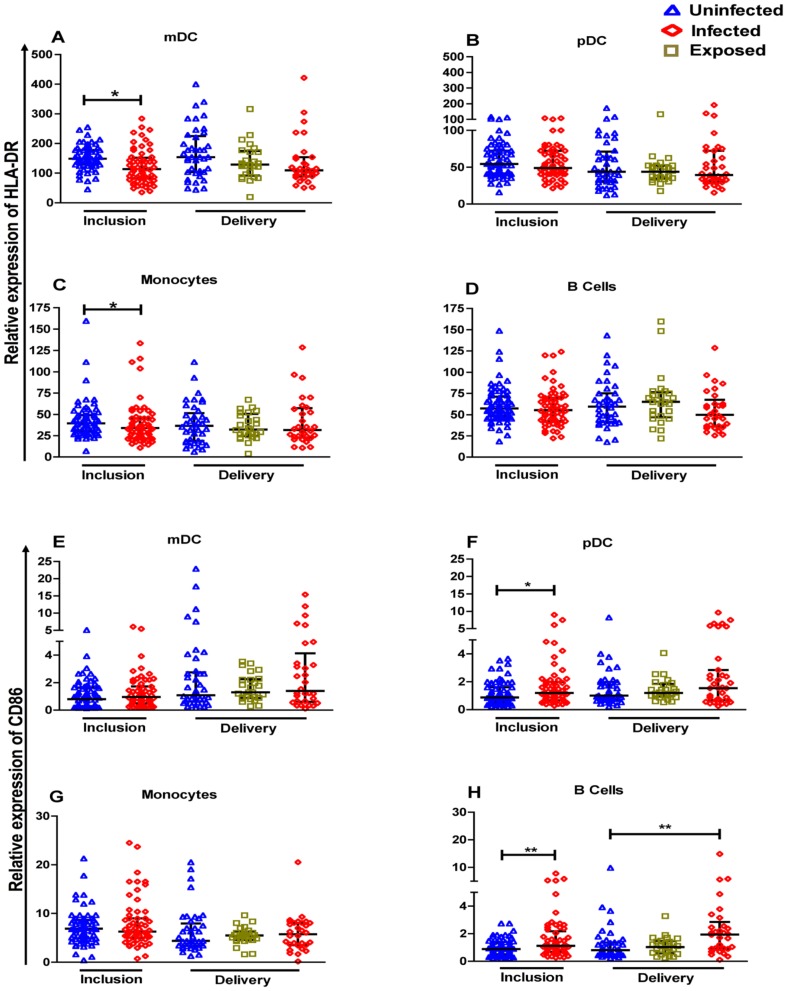
*P. falciparum* infection-related changes in activation status of antigen-presenting cells at inclusion and delivery in pregnant Beninese. Scatter plots include bars depicting medians with interquartile ranges of HLA-DR expression (A–D) and CD86 expression (E–H) on APC of 69 uninfected compared to 62 infected women at inclusion and of 47 uninfected, 27 exposed and 37 infected women at delivery. The statistical significance of differences in levels of expression were determined using the non-parametric Mann Whitney U test for data at inclusion combined with the Kruskall Wallis test for data at delivery. *p<0.05, **p<0.01, ***p<0.001.

Univariate analysis of the Beninese dataset included immunological variables as well as age, gestational age, *P. falciparum* infection status, and bednet possession. Figure 2 illustrates the flow cytometry-based characterization by phenotypic markers of the different populations of cells within PBMC that we identified for comparisons. A number of variables were found to display associations with *P. falciparum* infection detectable at inclusion (Table 2). These included, as outlined above, age, anaemia and bednet possession. Amongst the cellular phenotypes of antigen-presenting cells, the frequencies of mDC/pDC and monocytes did not differ according to infection status but the frequency of B cells was significantly higher in PBMC of infected versus uninfected women (Figure 3A–D). Within T cell subsets, the only difference found concerned the significantly lower frequency of Treg in PBMC of infected versus uninfected women (Figure 3E–H). With respect to activation status, HLA-DR expression on both mDC and monocytes was significantly lower whilst CD86 expression on both pDC and B cells was significantly higher in PBMC of infected versus uninfected women (Figure 4A–H).

All the variables displaying associations with infection in univariate analysis were subsequently included in multiple logistic regression analysis. The latter confirmed the independent association between maternal anaemia and infection (Table 3), but the association with bednet possession was lost. Variables that remained significantly different in PBMC of infected compared with uninfected women after multivariate analysis included the lower frequency of Treg, the higher frequency of B cells overall and the higher frequency of B cells expressing high levels of CD86 (Table 3). We next conducted a sub-analysis using ordered logistic regression that categorised women according to their parasite density as uninfected or with a parasitaemia above or below the infected group's median value. This revealed significant associations for the same set of variables as for infection alone, namely that higher parasitaemia loads were associated with younger age (OR 5.3, 95% CI 2.0–14.0; p<0.05), more frequent anaemia (OR 2.8, 95% CI 1.0–7.5; p<0.01), significantly fewer Treg (OR 3.8, 95% CI 1.6–9.2; p<0.01) and a significantly higher proportion of B cells expressing high levels of CD86 (OR 3.4, 95% CI 1.4–8.2; p<0.01). Linear regression analysis showed that higher parasitaemia loads were also associated with significantly reduced levels (p≤0.001 in both cases) of expression of both HLA-DR and CD86 on monocytes (data not shown).

**Table 3:** Multiple logistic regression analysis for independent associations of selected parameters with the presence of *P. falciparum* infection in the Benin sub-group at inclusion.

Variables	Odds Ratio	95% CI	p
% with hemoglobin <11 g/dl	4.2	1.5—11.7	<0.01
% aged ≤25 years	2.7	1.0—6.9	<0.05
% B cells (≥median)^a^	2.5	1.0—6.1	<0.05
% B cells expressing CD86^hi^ (≥median)^a^	3.3	1.3—8.1	= 0.01
% Treg (<median)^a^	3.1	1.3—7.7	= 0.01

a median value of uninfected group used for dichotomisation.

χ^2^ Hosmer-Lemeshow test (15ddl) = 13.9, p = 0.53.

Our overall analysis strategy comprised validation of the findings generated by the analysis of the Beninese dataset through identical analysis of the Tanzanian dataset. Using the latter, we therefore next conducted multiple logistic regression analysis in the same way as with the Beninese dataset, incorporating the same variables for assessment as a function of the presence or absence of *P. falciparum* infection. This revealed a significantly higher proportion of B cells with a high level of expression of CD86 (OR 13.8; IC 95% 1.6–120.6; p<0.05), and a significantly lower proportion of Treg (Treg/Teff ratio) (OR 8.4; IC 95% 1.2–57.5; p<0.05) in infected versus uninfected Tanzanian women at inclusion, confirming the results of analysis of the Beninese data.

Of note, we found no evidence of differences in the frequencies of T (CD4^+^/CD8^+^, Figure 3G, H), NK or NKT cells (data not shown) as a function of the presence or absence of *P. falciparum* infection at inclusion.

### P. falciparum infection-related changes in the peripheral blood mononuclear cell composition at delivery

Univariate analysis of the Benin dataset at delivery included the same set of variables as at inclusion. The analysis revealed differences with respect to a comparatively restricted number of variables, specifically concerning significantly higher frequencies of B cells and effector T cells (Teff) but lower frequencies of pDC in PBMC of infected versus uninfected women (Figures 3B,D,F & 4H). In multiple logistic regression analysis PBMC from infected women were shown to contain significantly more B cells that expressed higher levels of CD86, significantly fewer pDC, significantly more mDC expressing a low level of HLA-DR, and significantly more Teff cells compared to uninfected women (Table 4).

**Table 4:** Multiple logistic regression analysis for independent associations of selected parameters with the presence of *P. falciparum* infection in the Benin sub-group at delivery.

Variables	Odds Ratio	95% CI	p
% pDC (<median)^a^	2.3	1.0—5.7	= 0.06
% mDC expressing HLA-DR^low^ (<median)^a^	3.4	1.3—8.6	= 0.01
% B cells expressing CD86^hi^ (≥median)^a^	2.8	1.1—7.2	<0.05
% Teff (≥median)^a^	3.3	1.3—8.4	= 0.01

a median value of uninfected group used for dichotomisation.

We then performed a separate analysis combining the uninfected and ‘exposed’ groups into a single ‘non-infected’ group for comparison with the infected group that was itself dichotomised into those with either ‘low’ or ‘high’ parasite loads. Ordered logistic regression analysis in this case showed increasing parasite load to be associated with a significantly lower frequency of pDC (OR 4.8, 95% CI 1.4–16.9, p = 0.01), a non-significant trend for reduced expression of HLA-DR on mDC (OR 3.4, 95% CI 0.8–13.7, p = 0.09), and a significant increase in the frequency of Teff cells (OR 4.0, 95% CI 1.0–16.4, p = 0.05). Sample sizes here were too small to allow analysis by linear regression, and the small number of women included in the Tanzanian dataset at delivery (Table 2) precluded validation of the findings from the Beninese data.

As was the case for the analysis of data at inclusion, we found no evidence for differences in the frequencies of monocytes (Figure 3C), CD4^+^/CD8^+^ T cells (Figure 3G,H), NK or NKT cells (data not shown) as a function of the presence or absence of *P. falciparum* infection at delivery.

### Predictors of maternal anaemia

Of those mothers with relevant data available, 87/131 and 41/102 Beninese were anaemic (Hb<11 g/dL) at inclusion and delivery, respectively. As was the case for evaluation of association with infection, univariate analysis here included immunological variables as well as age, gestational age, *P. falciparum* infection status and bednet possession. Analysis at inclusion revealed the strongest (p<0.01) associations for anaemia and infection, age and reduced HLA-DR expression on mDC. Subsequent multivariate analysis showed independent associations between anaemia and infection, a gestational age at inclusion more than 17 weeks, and 3 separate PBMC-specific variables, namely (i) reduced HLA-DR expression on pDC, (ii) lower frequency of mDC, and (iii) higher expression of CD86 on monocytes (Table 5). Univariate analysis of data at delivery revealed only a single variable (increased frequency of Teff) associated with anaemia with a level of significance below the 5% level. In multivariate analysis, the increased frequency of Teff remained the only variable to display a significant association with anaemia at delivery (Table 6).

**Table 5:** Multiple logistic regression analysis for independent associations of selected parameters with the presence of anaemia in the Benin sub-group at inclusion.

Variables	Odds Ratio	95% CI	p
*P. falciparum* infection^a^	4.9	1.9—12.9	<0.01
% pDC expressing HLA-DR^low^ (<median)^a^	3.1	1.2—7.5	<0.05
% mDC (<median)^a^	2.9	1.2—7.1	<0.05
% Monocytes expressing CD86^hi^ (≥median)^a^	2.8	1.1—7.2	<0.05
Gestational age in weeks (>17 weeks)^a^	3.3	1.4—7.9	<0.01

a median value of non-anaemic group used for dichotomisation.

χ^2^ Hosmer-Lemeshow test (17ddl) = 7.91; p = 0.97.

**Table 6:** Multiple logistic regression analysis for independent associations of selected parameters with the presence of anaemia in the Benin sub-group at delivery.

Variables	Odds Ratio	95% CI	p
% mDC expressing HLA-DR^low^ (<median)^a^	2.5	0.9—6.8	= 0.07
% Teff (≥median)^a^	3.0	1.1—8.1	<0.05

a median value of non-anaemic group used for dichotomisation.

χ^2^ Hosmer-Lemeshow test (2ddl) = 0; p = 1.0.

In a prospective analysis, we examined the predictive value for anaemia at delivery of variables measured at inclusion. Apart from age, infection status, bednet possession and gravidity, the immunological variables retained for multivariate analyses as a function of univariate analyses were monocyte frequency, monocyte CD86 expression, B cell HLA-DR expression, Treg and Teff freqeuncy. The multivariate model showed that the risk of anaemia at delivery was associated with a higher frequency of monocytes overall and of monocytes expressing high levels of CD86 amongst PBMC at inclusion (Table 7).

**Table 7:** Prospective analysis of association between variables at inclusion and anaemia at delivery.

Variables	Odds Ratio	IC 95%	p
% Monocytes (>median)^a^	3.1	1.04—9.06	<0.05
% Monocytes expressing CD86^hi^ (≥median)^a^	3.2	1.04—9.73	<0.05
% B cells expressing HLA-DR^hi^ (≥median)^a^	2.6	0.88—7.46	= 0.08

a median value of non-anaemic group used for dichotomisation.

χ^2^ Hosmer-Lemeshow test (4ddl) = 0.91; p = 0.92.

## Discussion

The immunological study we present here constituted a part of our larger STOPPAM study. STOPPAM inevitably had a major impact on various parasitological and immunological outcomes in the mothers participating in it. This is due to both the active (scheduled ante-natal clinic visits) and the passive (unscheduled ‘emergency’ visits) modes of detection of infection integral to it, allied to the fact that curative anti-malarial treatment was given whenever *P. falciparum* infection was detected. In addition, the participants received intermittent preventive treatment with sulfadoxine-pyrimethamine (IPTp SP) on two occasions during pregnancy as per national policy in the two countries concerned. Any interpretation of our findings must therefore necessarily take into account these facts.

In the discussion that follows, we discuss the profiles of the different cell types we observed sequentially, focusing on those that our analysis revealed to vary significantly as a function of the presence of infection with *P. falciparum*, whilst also comparing and contrasting the profiles observed at inclusion versus delivery. It is of particular note that maternal anaemia was independently associated with *P. falciparum* infection present at inclusion but not at delivery. In this context, the women's infection histories, when allied to molecular genotypic characterisation of parasite isolates from them (Tuikue Ndam N., unpublished data), revealed that 90% of infections at delivery (29/32 women in the selected immunology sub-group for whom relevant data was available) were acquired within the preceding 4 weeks. This finding, coupled with the complete absence of malarial haemozoin pigment in placental biopsy samples from those with infections at delivery (Fievet N., unpublished data), leads us to conclude that the overwhelming majority of infections detected at delivery were acute i.e. recently acquired in nature. In contrast, we can make no similarly conclusive statement concerning the duration of *P. falciparum* infections detected at inclusion. The median gestational age at inclusion was 18 weeks, which allows a theoretical time-frame for acquisition of infections prior to inclusion of 2–3 weeks or, potentially, 1–4 months. Despite this possible range, for reasons outlined below, our conclusions are based on the assumption that a majority of the *P. falciparum* infections we detected at inclusion were in fact chronic rather than acute in nature i.e. the immunological assessments we made directly reflect the differing character of the infections at inclusion and delivery. In this context, it is also notable that we found gestational age-related changes to PBMC profiles that were independent of womens' *P. falciparum* infection status (Ibitokou S et al, manuscript submitted)

Compared to PBMC of uninfected women we found a significantly increased frequency of immature monocytes i.e. cells expressing reduced levels of both HLA-DR and the co-stimulatory molecule CD86 [22], in the PBMC of infected women at inclusion but not in those of infected women at delivery. The latter finding is consistent with that of an earlier study in which we showed that the expression of markers of activation on monocytes present in PBMC at delivery was similar, regardless of the presence or absence of infection [16]. Circulating immature monocytes are found during malaria attacks in children [23], and such cells are indicative of an on-going inflammatory response [22]. There is general consensus that monocytes form the principal component of the inflammatory response to *Pf*iE in the placenta [13], [24], [25]. Earlier studies, including our own, have shown that the monocytes present in infected placentas are activated, as reflected by upregulated expression of HLA-DR, CD86 and CCR5 [16], [26]. We therefore speculate that chronic on-going *P. falciparum* infections, as seen at inclusion of women into the current study, lead to a localized and persistent turnover of monocytes in the placenta requiring replacement from the bone marrow pool. By corollary, recently acquired (acute) infections, as found here at delivery, have not yet depleted the locally-available population sufficiently to require recruitment of immature cells from the bone marrow. In work to be reported elsewhere we show that the concentrations of the chemokines interferon-gamma-inducible protein (IP)-10, in particular, and monocyte chemotactic protein (MCP)-1 were elevated in the peripheral plasma of infected mothers, clearly indicating on-going monocyte chemoattraction (Boström S & Ibitokou S, unpublished data). Enhanced activity of various chemokines, including both IP-10 and MCP-1, is reported to be associated with placental infection by *P. falciparum* [13], [27]–[32]. Moreover, it is well-established that chemokines show specific binding affinities for glycosaminoglycans (GAG) [33]–[35], thereby establishing concentration gradients via which cells are recruited to sites of inflammation. The CSA expressed by syncytiotrophoblasts that acts as the receptor for *Pf*iE in the placenta is itself a GAG, and its potential interactions with chemokines thus clearly merit further research.

We found no significant infection-associated alterations to DC frequencies in PBMC at inclusion, although reduced expression of HLA-DR on mDC, possibly indicating functional impairment of this subset, was seen. Infection at delivery, on the other hand, was associated with loss of circulating pDC. These observations are entirely consistent with those reported in studies of DC from Senegalese mothers with *P. falciparum* infections at delivery [17]. They also accord well with DC profiles seen during malaria attacks in Kenyan children and, in the case of loss of pDC, in Thai adults [23], [36], [37]. It is known that pDC express CXCR3 [33], a receptor with specificity for the chemokines monokine-induced by IFN-γ (MIG) and IP-10, as well as CCR2, the ligand for which is MCP-1. MIG, IP-10 and MCP-1 were all present at significantly higher levels in the peripheral plasma of infected women (Boström S & Ibitokou S, unpublished data), potentially providing a stimulus for migration of DC from the peripheral circulation to lymphoid tissues at the site of infection. A prominent, potentially pivotal role of pDC in the evolution of immune responses to malarial parasites, as suggested recently [38], is not evident from our data, but more detailed investigation would be necessary to clarify this point.

The increased frequency in PBMC of B cells in association with *P. falciparum* infections - including higher proportions expressing elevated levels of the activation marker CD86 - was a consistent finding at both time-points in the present study. This is indicative of the strong involvement of B cells in the placental inflammation associated with *P. falciparum* infections during pregnancy [13]. Overall B cell frequencies during acute *P. falciparum* infections in children are reported to be either unchanged [39] or increased, as seen here [40]. Since B cells are known to express toll-like receptor (TLR) 9, upregulation of CD86 on B cells may result from the same parasite antigen-mediated pathway reported for pDC [36]. As is also the case for pDC mentioned above, B cells are known to express the chemokine receptor CXCR3, and they could thus be assumed to be responding in the same way to the elevated concentrations of the MIG and IP-10 chemokines present in the plasma of infected women. The CXCL13-CXCR5 axis of B cell recruitment has also been reported to play a prominent role in this context [13]. A plausible conclusion from our own and others data would therefore be that B cell responses, and by inference antibody production and secretion locally in the placenta, are primary components of the maternal immune response to infection with *P. falciparum* during pregnancy. Such B cell responses are thought to be activated in a T cell-independent manner [13]. A potent non-specific T cell-independent activator of B cells that is able to bind multiple molecules, including IgM, has been identified in *P. falciparum* [41], [42], and VAR2CSA, the parasite-derived ligand for CSA, displays a similar capacity for non-specific binding of IgM [43]. Given the candidacy of VAR2CSA as a vaccine to prevent malaria in pregnancy, priority should be given to fully characterizing its domain(s) potentially mediating this particular property and, equally, to determine whether it extends to non-specific activation of B cells. Activated B cells, furthermore, are purported to play a prominent role in the regulation of human DC responses, particularly on the monocyte-mDC maturation axis [44]. The importance of elucidating whether such interactions occur in the context of PAM and/or a candidate vaccine thus seems clear enough.

The changes in Treg and Teff profiles that were associated with *P. falciparum* infections differed at inclusion and delivery. These changes are of particular interest given the potentially pivotal role Treg may play in determining the outcome of infection [45], [46]. At inclusion, Treg were found at a significantly lower frequency in PBMC of infected women compared to uninfected women, whilst at delivery the frequency of Teff relative to Treg was significantly higher. Reduced frequencies of Treg in PBMC have not been reported during *P. falciparum* infections, in fact they have been reported to be elevated during infections of non-pregnant Africans [46]. Our observation is nevertheless consistent with reports in the literature of ‘sequestration’ of Treg during non-plasmodial infections that display tissue- or organ-specificity. During pregnancy, *P. falciparum* displays a clear tissue-specificity, localising to the placenta, that is in marked contrast to the normally systemic distribution of *Pf*iE in non-pregnant hosts. The apparent ‘loss’ of Treg from PBMC of infected women that we observed at inclusion thus probably reflects their migration to the infected placenta, mediated, as for monocytes and other cell types, by the increased concentrations of chemokines such as IP-10. The anti-inflammatory functions of Treg involve production of, for example, cytokines such as IL-10, which could at least partially explain the increased concentrations of IL-10 we observed in peripheral plasma in parallel with the different chemokines mentioned earlier (Boström S & Ibitokou S, unpublished data). The impact that any of the infection-mediated changes in circulating Treg observed here may have on the reported gestational age-related fluctuations in the frequency of Treg in PBMC during normal, healthy pregnancies remains to be determined [4], [47], [48]. Whether such fluctuations occur during ‘normal’ healthy pregnancies in African populations in any case requires confirmation. The increased frequency of Teff associated with infections at delivery but not with those at inclusion may be a further reflection of the acute nature of the infections present at delivery, whilst the generalized lymphopenia that is a classical feature of symptomatic *P. falciparum* was not seen at either time-point, a finding that may reflect both the asymptomatic character and the comparatively low-level parasitaemias of the infections.

In the specific context of anaemia during pregnancy, the associations with PBMC profiles we observed at inclusion and delivery involved DC/monocytes and effector T cells, respectively. The aetiology of anaemia during pregnancy is acknowledged to be complex, but one plausible explanation for those differences might be, as suggested by others [49], the relationship between the time-course of inflammatory responses - exemplified here in particular by the increased frequency of activated (CD86^high^) monocytes associated with anaemia at inclusion - and *P. falciparum* infection, in the sense that inflammation and hence anaemia will tend to ‘lag behind’ infection. In the same context, we interpret the increased frequency of Teff in PBMC at delivery, associated with both infection and anaemia, as an indication of the presence of T cell subsets (e.g. effector memory cells) that are able to respond rapidly to infection. The contrasting association between anaemia and reduced HLA-DR expression on DC, on the other hand, might plausibly reflect the known suppressive effects of IL-10 - present at significantly higher levels in the plasma of infected women - in this context [50].

In summary, we have identified a number of features of PBMC associated with *P. falciparum* infection and with anaemia during pregnancy. The striking association between infection and increased B cell frequencies in PBMC, irrespective of gestational age, emphasizes the role such cells appear to have in the context of infection-induced placental inflammation. Our data also serve to clearly highlight the prominent role played by monocytes in that inflammatory response. They further suggest that Treg provide a balancing anti-inflammatory response through, we assert, their migration to the placenta as the primary site of *P. falciparum* infection during pregnancy. That Treg may respond and be reallocated in such a way is entirely consistent with current knowledge of the pathology-limiting controlling role such cells are thought to play during tissue-specific infections.
